# Observable Metabolites and Metabolomic Sampling Protocols for Managed African Savanna Elephant (*Loxodonta africana*) Whole Blood Using H-NMR Spectroscopy

**DOI:** 10.3390/metabo12050400

**Published:** 2022-04-28

**Authors:** Jordan Wood, David R. Morgan, Kimberly Ange-van Heugten, Maria Serrano, Larry J. Minter, Vivek Fellner, Michael K. Stoskopf

**Affiliations:** 1 Department of Animal Science, North Carolina State University, Raleigh, NC 27607, USA; jnwood@ncsu.edu (J.W.); kdange@ncsu.edu (K.A.-v.H.); vivek_fellner@ncsu.edu (V.F.); 2 Environmental Medicine Consortium, North Carolina State University, Raleigh, NC 27607, USA; drmorgan@mail.com (D.R.M.); meserran@ncsu.edu (M.S.); jb.minter@nczoo.org (L.J.M.); 3 Department of Clinical Sciences, College of Veterinary Medicine, North Carolina State University, 1060 William Moore Dr, Raleigh, NC 27607, USA; 4 North Carolina Zoo, 4401 Zoo Pkwy, Asheboro, NC 27205, USA

**Keywords:** proton-NMR, metabolomics, African elephant

## Abstract

We used nuclear magnetic spectroscopy (NMR) to evaluate the metabolomics of heparinized whole blood drawn from six African savanna elephants (*Loxodonta africana*) maintained on a well characterized diet. Whole blood samples obtained under behavioral restraint, then quickly frozen in liquid nitrogen, were stored at −80 °C until analysis. Frozen samples were thawed under controlled conditions and extracted with methanol and chloroform to separate the polar and non-polar metabolites. We identified 18 polar metabolites and 14 non-polar lipids using one-dimensional (1D) and two-dimensional (2D) NMR spectra. Despite unexpected rouleaux formation in the thawed frozen samples, spectra were consistent among animals and did not vary dramatically with age or the sex of the animal.

## 1. Introduction

Basic challenges for the conservation of the seasonally migratory African savanna elephants (*Loxodonta africana*) have been long identified [1]. Considerable research into the ecology and behavior of these charismatic mega vertebrates have documented many concerns for the long-term preservation of the species. Political fragmentation of habitats, particularly in elephant migratory routes, fencing, and a variety of deleterious human–elephant interactions contribute to concerns about a bleak future for the species, sometimes referred to as an ecosystem engineer [2,3,4,5,6,7,8].

Physiologic studies of the remarkably adaptive African elephant have been more limited than ecological studies. Studies have focused on fecal glucocorticoids and salivary progesterone metabolites for tracking stress responses and ovarian cycles, useful for understanding reproductive efficiency [9,10,11,12,13]. These studies are generally conducted on captive or semi-captive animals. Studies of free ranging African elephant physiology have largely been limited to routine serum enzyme and electrolyte concentrations [14] and studies of concentrations of immune system biomarkers [15]. Relatively little has been done to evaluate elephant metabolism using the emerging field of metabolomics.

Metabolomic studies quantify the small molecule content of a sample, providing a much more dynamic evaluation of metabolism than routine clinical chemistries. These studies can be conducted using different analytical modalities, but nuclear magnetic resonance spectroscopy (NMR) provides better quantitative information on metabolite concentrations [16]. Studies of humans and domestic species have demonstrated that metabolomics can offer useful information on health [17,18,19,20,21,22,23,24]. The use of NMR metabolomics for health applications is still novel and the establishment of techniques and baseline information is needed to support more complex evaluations of wildlife health.

Relatively recent advances in metabolomics methods have allowed the evaluation of whole blood as a representative sample [22]. Whole blood offers the advantage of simpler sample handling at collection, making more rapid quenching of metabolic activity possible. It also offers the advantage of including intracellular metabolite data in the assessment [16].

Baseline data on animals maintained in known environments and diets facilitates interpretation of more advanced studies exploring the physiologic impacts of environmental stressors or disease. In this study, whole blood from African savanna elephants fed a well characterized diet was used to evaluate metabolites by NMR.

## 2. Results

**Figure 1:**
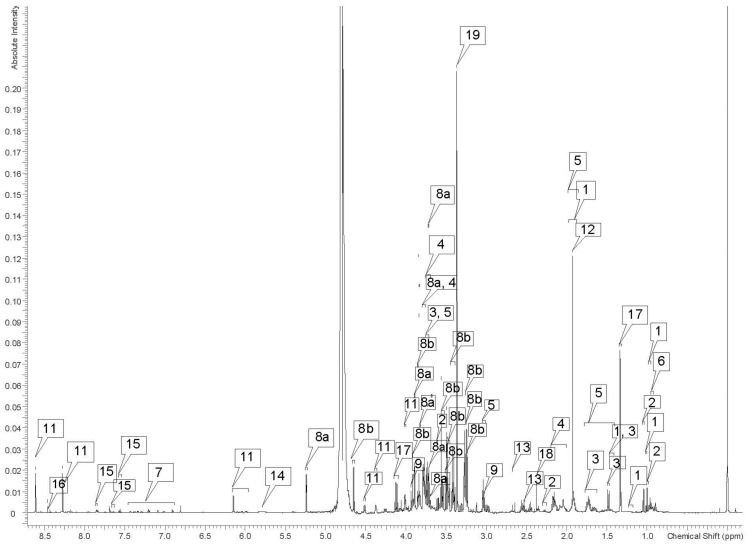
Representative 1D 600 MHz proton NMR spectra of the polar extract of whole blood from an 18-year-old female African savanna elephant (*Loxodonta africana*). The labeled peaks are as follows: (1) isoleucine, (2) valine, (3) alanine, (4) leucine, (5) lysine, (6) glycine, (7) tyrosine, (8a) α-glucose, (8b) β-glucose, (9) creatine, (10) creatinine ^§^, (11) adenosine triphosphate, (12) acetate, (13) citrate, (14) urea, (15) benzoate, (16) formate, (17) lactate, (18) succinate, (19) methanol. ^§^ Singlet peaks at 3.03 and 4.06, visible on expanded spectrum, not flagged due to label congestion in the region.

**Figure 2:**
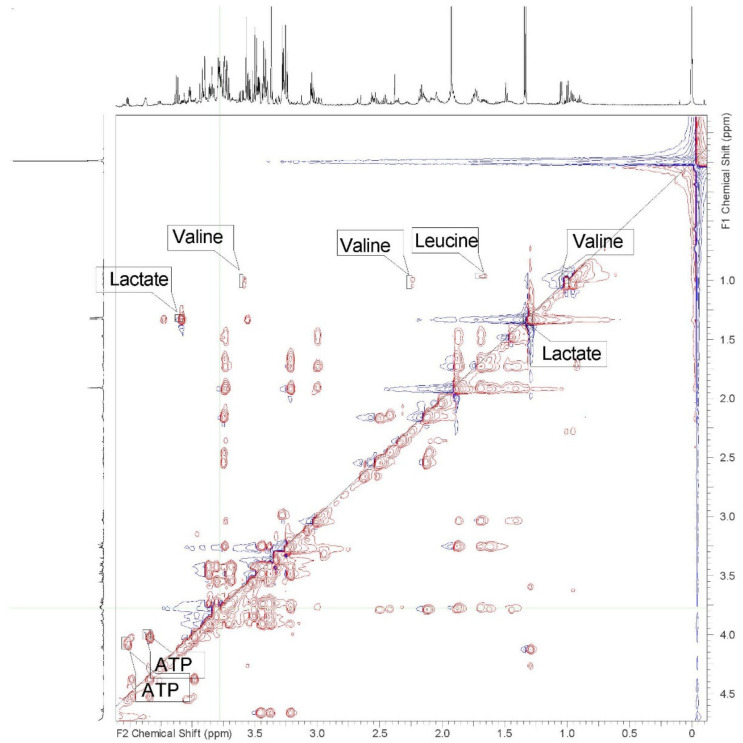
2D TOCSY proton NMR spectra of the polar extract of whole blood from an 18-year-old female African savanna elephant (*Loxodonta africana*). Representative labeled peaks are valine, lactate, leucine, and adenosine triphosphate (ATP).

Despite using lithium heparin as an anticoagulant, all six samples demonstrated rouleaux formation when thawed for preparation and extraction. This was most dramatic in the sample from the oldest male (46 years) requiring the use of 100 μL less blood in the processing of the sample. Spectral data was similar for all animals, with consistent chemical shifts for all identified peaks and metabolites in the polar samples. (See Supplemental Data to view all spectra.) Differences in the concentrations of metabolites were minor and well within the range of variation expected, due to small variations in the time of day of sampling. No differences attributable to sex or age were recognized in polar samples. Nineteen metabolites were identified in 1D spectra and verified with 2D spectra in the polar samples (Table 1 and Figure 1 and Figure 2). Some peaks were too small or overlapped to be clearly identified and verified using 1D and 2D spectra, but peaks could be correlated using correlated spectroscopy (COSY), total correlation spectroscopy (TOCSY), heteronuclear-multiple bond correlation (HMBC), and heteronuclear single quantum coherence (HSQC) (Table 1).

**Figure 3:**
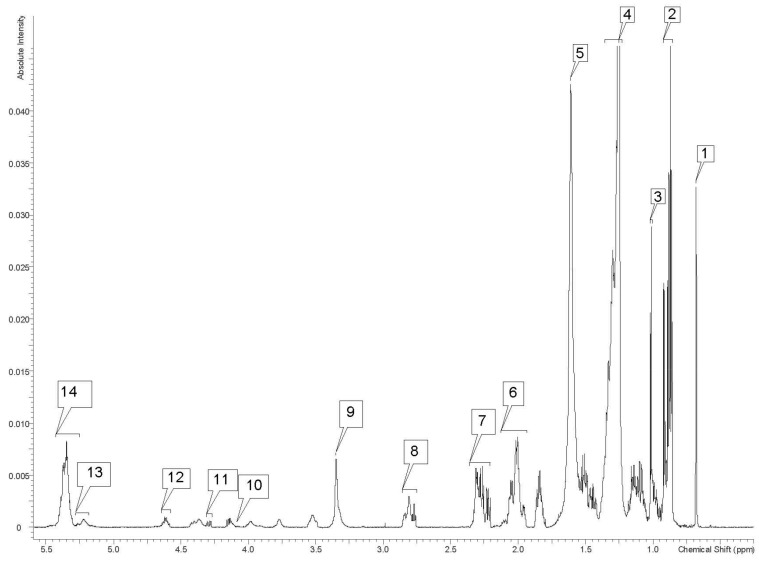
1D 600 MHz proton NMR spectrum of non-polar extract of whole blood from a 47-year-old male African savanna elephant (*Loxodonta africana*). The labeled peaks are as follows: (1) cholesterol methyl of C18, (2) methyls of saturated and monosaturated omega-9 and or omega-7 chains and unsaturated omega-6 acyl groups, (3) C19 methyl of free cholesterol, (4) CH_2_ in fatty acyl groups, (5) CO-CH_2_-CH_2_ in fatty acyl chain, in a complex peak that overlaps with water, (6) double bonds in fatty acyl chains except for DHA acyl groups, (7) ethylene methyl in fatty acyl chain except for DHA acyl group, (8) double bonds in fatty acyl chain of 18:2, 20:4, and 22:6, (9) phosphatidylcholine, (10) carbon 1 and 2 in glycerol backbone of triglycerides and phospholipids, (11) carbon 1 and carbon 3 in glycerol backbone of triglycerides, (12) esterified cholesterol, (13) glycerol groups in the backbone of triglycerides and phospholipids, (14) double bonds in fatty acyl chains.

Non-polar samples were also consistent for all elephants. Non-polar fractions are listed in Table 2 and represented by Figure 3, as well as the processed 1D and 2D spectra for all elephants which are included in the Supplementary Data.

## 3. Discussion

The use of 1D and 2D ^1^H NMR methods allowed identification of 18 metabolites in the aqueous fractions, and 14 metabolites in the lipophilic fractions of the blood of the elephants in our study. The lipophilic fraction is frequently not described in ^1^H NMR studies of animals. Despite the challenges of identifying specific metabolites in lipophilic fractions using ^1^H NMR, this information can provide valuable insights into the metabolism of the species being studied.

An unexpected challenge to this study was the discovery of rouleaux formation, present in the thawed samples despite the addition of anticoagulant prior to rapid freezing. This has not been a problem in work with similarly treated blood samples of other species examined in our laboratory, but is known in elephant blood samples treated similarly when drawn for routine hematology and plasma or serum chemistry panels. Early studies of the blood coagulation of Asian elephants (*Elephas maximus*) describe elephant blood as clotting quickly and determined the presence of higher concentrations of clotting factors and faster partial thromboplastin times than found in human blood [25], but later work, also looking at Asian elephants, found plasma levels of specific coagulation factors to be like those in human plasma with the exception of FVIII:C, which was elevated [26]. The activated partial thromboplastin time found in those studies was three-fold those found in the earlier study and longer than found for human reference plasma. The rouleaux formation we observed may have been exacerbated by the rapid transfer of blood samples into liquid nitrogen. Other blood samples collected from the elephants the same day at the same time using the same anticoagulant treated vials were processed for routine complete blood counts without noted problem. Waiting 10 min before plunging the samples into liquid nitrogen would not appreciably change the metabolite concentrations in the sample and might be considered in the future [27]. When rouleaux formation was observed in the laboratory after the samples were thawed, considering the objective of the study, it would have been better to sonicate or otherwise homogenize the sample as is done to prepare solid tissues for extraction. The spectra obtained were very consistent across samples, but a portion of intracellular metabolites may have been sequestered in the white cells potentially trapped in the gelled rouleaux formation. On the other hand, erythrocytes, and presumably leukocytes, appeared to have been ruptured in the freeze/thaw process, likely reducing the impact of the rouleaux formation on metabolite concentrations in the liquid portion of the sample. It is also possible that the concentrations of the metabolites we detected did not vary a great deal between the intracellular and extracellular compartments.

One non-polar sample, apparently contaminated with water and methanol, likely was the result of incomplete dry down under nitrogen, as the spectrum was otherwise not dissimilar to those of the other five animals. We cannot rule out a possible pipetting error but failure to fully complete the drying step prior to reconstitution of the sample for placement in the magnet is most likely, considering the similar peak heights in the 1D spectra of this sample.

The metabolite concentrations were surprisingly similar in this study. This is likely due to the advantage of the elephants all being maintained on the same carefully managed diet (see materials and methods), and the drawing of blood samples within a narrow time of day window without the need for restraint. Elephants are quite able to adapt to varied diets and future studies might explore the metabolics of such adaptations. It would also be of great interest to explore the impact of the time of day of sampling for animals on different diets. This study provides preliminary information on the expected metabolites in healthy elephants. It suggests that no large differences should necessarily be expected due to age or the sex of the animal. Perturbations in the metabolome of elephants may be valuable markers for environmental, social and other stressors, or various forms of disease that would be expected to impact the metabolome.

## 4. Materials and Methods

### 4.1. Animals and Sample Collection

Six healthy African elephants (two males, four females) ranging from 18 to 46 years of age were involved in this study with the approval of the North Carolina (NC) Zoo Research Committee. Basic health parameters (hematology and serum chemistry) are maintained for these animals in the Species360 Zoological Information Management System (ZIMS.species360.org). These animals were maintained on a daily diet that included an estimated 27–59 kg exhibit pasture (fescue grass (*Festuca arundinacea*), annual ryegrass (*Lolium multiflorum*), and Bermuda grass (*Cynodon dactylon*) some white clover (*Trifolium repens*)) during the day, an average of 11 kg of various browse plant species overnight, 6 kg produce enrichment items, 132–155 kg timothy hay (*Pheleum pratense*), and 3–4 kg of Mazuri^®^ Hay Enhancer™ for females and males, respectively [28]. The analysis of the diet of these animals including various browse species fed, are more fully reported by Wood et al. [28]. Table 3 lists the most commonly provided species of browse and the percentage of the time they are presented over a year’s duration.

Blood samples were collected from a posterior auricular vein using a 21-gauge butterfly catheter and lithium heparin coated tubes (BD Vacutainer^®^ Becton, Dickinson and Company, Franklin Lakes, NJ, USA) followed by aliquoting into 1 mL cryovials (Thermo Scientific™ Nalgene™ General Long-term Storage Cryogenic Tubes; Thermo Fisher Scientific, Hampton, NH, USA) and immediate quenching with liquid nitrogen, in August of 2020. Samples were stored at −80 °C at the NC Zoo Veterinary Hospital until transportation on dry ice to the NC State University Center for Marine Sciences and Technology (CMAST) for further storage at −80 °C until analysis.

### 4.2. Sample Preparation

Whole blood samples frozen at −80 °C were thawed on ice. Despite the use of lithium heparin, rouleaux formation was present in all six samples. A total of 800 μL of the blood was combined stepwise with methanol and chloroform in a 1:2:2 ratio, vortexing for 30 s between additions. One sample (male, 46 years) had greater rouleaux formation than the rest of the elephants and only 700 μL of blood could be used. After sonication at 4 °C for 2 min, samples were incubated for 20 min at −20 °C in a traditional freezer. Then, samples were sealed with parafilm and centrifuged at 4 °C and 1000 RCF for 30 min to separate the samples into polar and non-polar fractions. Several samples were re-centrifuged at 1500 RCF to achieve better separation of the phases. Both phases of all samples were individually dried by rapid evaporation with nitrogen in a 25 °C water bath. Non-polar samples were dry within 30 min. Polar samples required 4–6 h to dry. Dried polar samples were reconstituted in 500 μL of a 7.4 pH phosphate buffer and 200 μL of D_2_O containing 0.05% TSP (chemical shift standard) and transferred to 5 mm NMR tubes for NMR analysis. Non-polar samples were reconstituted with 700 uL of CDCl_3_ with 0.03% tetramethylsilane (TMS) (Sigma-Aldrich Co., St. Louis, MO, USA), which was used as an internal standard, then transferred to 5 mm NMR tubes for analysis.

### 4.3. NMR Data Collection

A Varian Inova 600 MHz two-channel spectrometer with a variable temperature unit was used in conjunction with a Varian two-channel inverse detection probe with z-gradients and a variable temperature capacity to collect NMR data. We collected 1D spectra and COSY and TOCSY data with the proton channel and the HSQC and HMBC used ^1^H and ^13^C channels. The samples were allowed at least 5 min to equilibrate at the probe temperature of 25 °C. After this time, the probe was tuned, shimmed, and the 90-degree pulse measured. Utilizing the new pulse information, several short proton spectra with a 2 s relaxation delay were acquired with different pulse angles to assure the spectra would be quantitative. The water suppression technique was pre-saturation.

Furthermore, 1D analyses of polar fractions were run at a frequency of 599.69 MHz and temperature of 29 °C due to room cooling difficulties, with a sweep width of 7200 Hz. The total acquisition time was 45 min and 25 s with 256 scans. The non-polar fractions were analyzed at the same frequency and sweep width but at 25 °C, 32 scans, and the total acquisition time was 7 min and 46 s. In addition, 2D spectra including COSY, TOCSY, HSQC, and HMBC were acquired for one polar sample and one non-polar sample for further identification of the metabolites present. Polar COSY was run for 3 h with a sweep width of 7200 Hz and 128 increments, TOCSY was run for 3 h and 30 min with a sweep width of 7200 Hz and 256 increments, HSQC was run for 12 h and 30 min with a sweep width of 7200 Hz and 256 increments, and HMBC was run for 21 h and 30 min with a sweep width of 7200 Hz and 128 transients. Non-polar COSY was run for 21 min with a sweep width of 7200 Hz and 128 increments, TOCSY was run for 2 h and 30 min with a sweet width 7200 Hz and 256 increments, HSQC was run for 11 h with a sweep width of 9608 Hz and 256 increments, and HMBC was run for 11 h and 20 min with a sweep width of 7200 Hz and 256 increments.

### 4.4. Data Analysis

NMR data was examined using an ACD labs 12.0 1D NMR processor (Advanced Chemistry Development, Toronto, Ont, Canada). The spectral processing protocol used included zero-filling each spectrum to 16,000 points and Fourier transformation. Automatic phasing and baseline adjustments were used with additional adjustments to baseline by hand for polar spectra. All spectra were then referenced to the TSP peak at 0 ppm (Hz/MHz). Metabolites were also identified using the Human Metabolome Database (HMDB) and additional literature sources for comparisons. The 2D spectra collected verified identifications from the HMDB and the previous literature. Unknown peaks that could not be accurately identified could be hypothesized to be aromatic rings through TOCSY and HMBC. The HMBC spectra also helped with identifying metabolites with acid groups such as the amino acids or lactate. The TOCSY spectra provided clear connections to determine α- and β-glucose peaks despite significant overlap on the 1D spectra.

## Figures and Tables

**Table 1:** Polar metabolites identified in the whole blood of African savanna elephants (*Loxodonta africana*) housed at the North Carolina Zoo in Asheboro, NC, USA, and their respective chemical shifts (ppm = Hz/MHz).

Label on Figure 1	Metabolite	^1^H Chemical Shifts (ppm)
**Amino Acids**		
1	Isoleucine	0.95 (t), 1.01 (d), 1.46 (u), 1.95 (u), 3.68 (u)
2	Valine	0.99 (d), 1.05 (d), 2.27 (u), 3.61 (d)
3	Alanine	1.47 (d, u), 3.79 (u)
4	Leucine	0.96 (d), 1.71 (s), 3.74 (u)
5	Lysine	1.45 (u), 1.50 (u), 1,72 (u), 1.93 (u), 3.03 (u), 3.73 (u)
6	Glycine	3.56 (s)
7	Tyrosine	6.90 (d), 7.20 (t, d)
**Energy Compounds**		
8α	α-glucose	3.70 (s), 3.71 (s), 3.73 (s), 3.74 (s), 3.75 (s), 3.78 (u), 3.82 (d), 3.84 (s), 3.89 (d), 5.24 (d)
8β	β-glucose	3.24 (s), 3.25 (s), 3.27 (s), 3.42 (m), 3.47 (m), 3.48 (s), 3.50 (s), 3.51 (s), 3.54 (dd), 3.85 (s), 3.86 (d), 3.91 (d), 4.65 (d)
9	Creatine	3.02 (s), 3.93 (s)
10	Creatinine	3.03 (s), 4.06 (s)
11	Adenosine triphosphate	4.02 (dd), 4.37 (u), 4.51 (dd), 6.15 (d, d, u), 8.27 (s), 8.61 (s)
**Organic Acids**		
12	Acetate	1.92 (s)
13	Citrate	2.55 (d), 2.66 (d)
14	Urea	5.79 (broad)
15	Benzoate	7.56 (t), 7.64 (t), 7.85 (d)
16	Formate	8.46 (s)
17	Lactate	1.33 (d), 4.12 (q)
18	Succinate	2.38 (s)
**Other**		
19	Methanol (residual)	3.36 (s)

**Table 2:** Non-polar lipid metabolite fractions identified in the whole blood of African savanna elephants (*Loxodonta africana*) housed at the North Carolina Zoo in Asheboro, NC, USA, and their respective chemical shifts (ppm = Hz/MHz).

Label on Figure 3	Lipid Metabolite Fraction	^1^H Chemical Shifts (ppm)
1	Cholesterol -CH_3_ C18	0.68
2	-CH_3_ (saturates, monosaturates w-9 and/or w-7, unsaturates w-6 acyl groups)	0.86–0.92
3	C19 -CH_3_ in free cholesterol	1.00–1.02
4	-(CH_2_)n fatty acyl group	1.23–1.36
5	-CO-CH_2_CH_2_ in fatty acyl chain	1.61
6	-CH_2_-CH=CH- (acyl group except for -CH_2_- of doxosahexaenoic acid (DHA) in β position related to carbonyl group) -CH_2_HC= in fatty acid acyl chain: 18:1, 18:2/20:4	1.94–2.13
7	-OCO-CH_2_- (acyl group except for DHA acyl group) -CO-CH_2_- in fatty acyl chain	2.20–2.35
8	-CHCH_2_CH= in fatty acyl chain: 18:2, 20:4/22:6	2.75–2.86
9	-N(CH_3_)_3_ (phosphatidylcholine)	3.35
10	>C_1_H_2_/C_2_H_2_ in glycerol backbone of triglyceride (TG) and phospholipids (PL)	4.12–4.17
11	>C_1_H_2_/C_3_H_2_ in glycerol backbone of TG	4.27–4.31
12	−3CH esterified cholesterol	4.58–4.65
13	-CHOCOR (glycerol group) -C_2_H in glycerol backbone of PL and TG	5.19–5.2
14	-CH=CH- in fatty acyl chain	5.25–5.43

1.310, 1.6253, 1.84, and 2.30, and 3.52 ppm are connected according to TOCSY spectrum; most likely associated with a fatty acyl chain. Multiplet at 5.26 ppm correlates with sharp peaks at 4.29 and 4.15 on COSY and TOCSY spectra and is predicted to be the second glycerol carbon of a TG; broad peak at 5.21 correlates with a doublet at 4.41, a broad peak at 4.12, and a broad peak at 3.97 according to the TOCSY spectra and is predicted to be the second glycerol carbon of a PL. Broad, overlapping multiplet at 5.35 ppm is from several double bonds on different molecules on long chains; correlations were found on COSY, TOCSY, HMBC, and HSQC spectra; COSY and TOCSY were correlated with peaks at 2.81 and 2.0—the 2.0 correlation is associated with protons on the carbon adjacent to the double bond, and the 2.81 is associated with protons on a carbon between two double bonds.

**Table 3:** Browse species most frequently fed to six North Carolina Zoo African elephants. (*Loxodonta africana*) and relative frequency of feeding over one year.

Browse Species	Relative Frequency Fed (per 100 Feedings)
Sweet Gum (*Liquidambar styractiflua*)	100
Wax Myrtle (*Morella cerifera*)	83
Mixed sp. Bamboo (*Phyllostachys sp.*)	81
Tulip Poplar (*Linodendron tulipifera*)	72
Thorny Elaegnus (*Elaeagnus pungens*)	70
Arundo (*Arundo donax*)	50
Willow Oak (*Quercus phellos*)	46
Pine (*Pinus sp.*)	41
Russian Olive (*Eleagnus angustifolia*)	36
Mimosa (*Albizia julibrissin*)	33
Dogwood (*Corus florida*)	26
Sugar Maple (*Acer saccharum*)	25
Contorted Mulberry (*Morus bornbycis*)	23
Pignut Hickory (*Carya glabra*)	20
Grapevine (*Vitis sp.*)	20
Chestnut Oak (*Quercus montana*)	18

Data extracted from Wood et al., 2020 [28].

## Data Availability

The data presented in this study are available in article and Supplementary Materials.

## Supplementary Materials

The following are available online at https://www.mdpi.com/article/10.3390/metabo12050400/s1, Figure S1: 1-D NMR Proton spectrum from 0.0 to 2.7 ppm of polar extract of whole blood from an 18 year old female African savanna elephant, Figure S2: 1-D NMR Proton spectrum from 2.8 to 5.6 ppm of polar extract of whole blood from an 18 year old female African savanna elephant, Figure S3: 1-D NMR Proton spectrum from 5.7 to 8.6 ppm of polar extract of whole blood from an 18 year old female African savanna elephant, Figure S4: 1-D NMR Proton spectrum of polar extract of whole blood from a 39 year old female African savanna elephant, Figure S5: 1-D NMR Proton spectrum of polar extract of whole blood from a 33 year old female African savanna elephant. Figure S6: 1-D NMR Proton spectrum of polar extract of whole blood from a 37 year old male African savanna elephant, Figure S7: 1-D NMR Proton spectrum of polar extract of whole blood from a 42 year old female African savanna elephant, Figure S8: 1-D NMR Proton spectrum of polar extract of whole blood from a 47 year old male African savanna elephant, Figure S9: 2-D NMR HSQC spectrum of polar extract of whole blood from an 18 year old female African savanna elephant, Figure S10: 2-D NMR HMBC spectrum of polar extract of whole blood from an 18 year old female African savanna elephant, Figure S11: 2-D NMR COSY spectrum of polar extract of whole blood from a 38 year old female African savanna elephant, Figure S12: 1-D NMR Proton spectrum from 0.0 to 2.5 ppm of non-polar extract of whole blood from a 47 year old male African savanna elephant, Figure S13: 1-D NMR Proton spectrum from 2.5 to 5.5 ppm of non-polar extract of whole blood from a 47 year old male African savanna elephant, Figure S14: 1-D NMR Proton spectrum of non-polar extract of whole blood from a 39 year old female African savanna elephant, Figure S15: 1-D NMR Proton spectrum of non-polar extract of whole blood from a 32 year old female African savanna elephant, Figure S16: 1-D NMR Proton spectrum of non-polar extract of whole blood from a 37 year old male African savanna elephant, Figure S17: 1-D NMR Proton spectrum of non-polar extract of whole blood from a 42 year old female African savanna elephant, Figure S18: 1-D NMR Proton spectrum of non-polar extract of whole blood from an18 year old female African savanna elephant, Figure S19: 2-D NMR HMBC spectrum non-polar extract of whole blood from a 47 year old male African savanna elephant, Figure S20: 2-D NMR HSQC spectrum non-polar extract of whole blood from a 47 year old male African savanna elephant, Figure S21: 2-D NMR TOCSY spectrum non-polar extract of whole blood from a 47 year old male African savanna elephant, Figure S22: 2-D NMR COSY spectrum non-polar extract of whole blood from a 47 year old male African savanna elephant, Table S1: Description of non-polar lipid metabolite fractions identified in spectrum of whole blood from a 47 year old male African savanna elephant.
