# Supplementary figures and images for: Observable Metabolites and Metabolomic Sampling Protocols for Managed African Savanna Elephant (Loxodonta africana) Whole Blood Using H-NMR Spectroscopy
Source: Metabolites. 2022 Apr 28;12(5):400. doi: 10.3390/metabo12050400 (PMC9143938; doi:10.3390/metabo12050400)

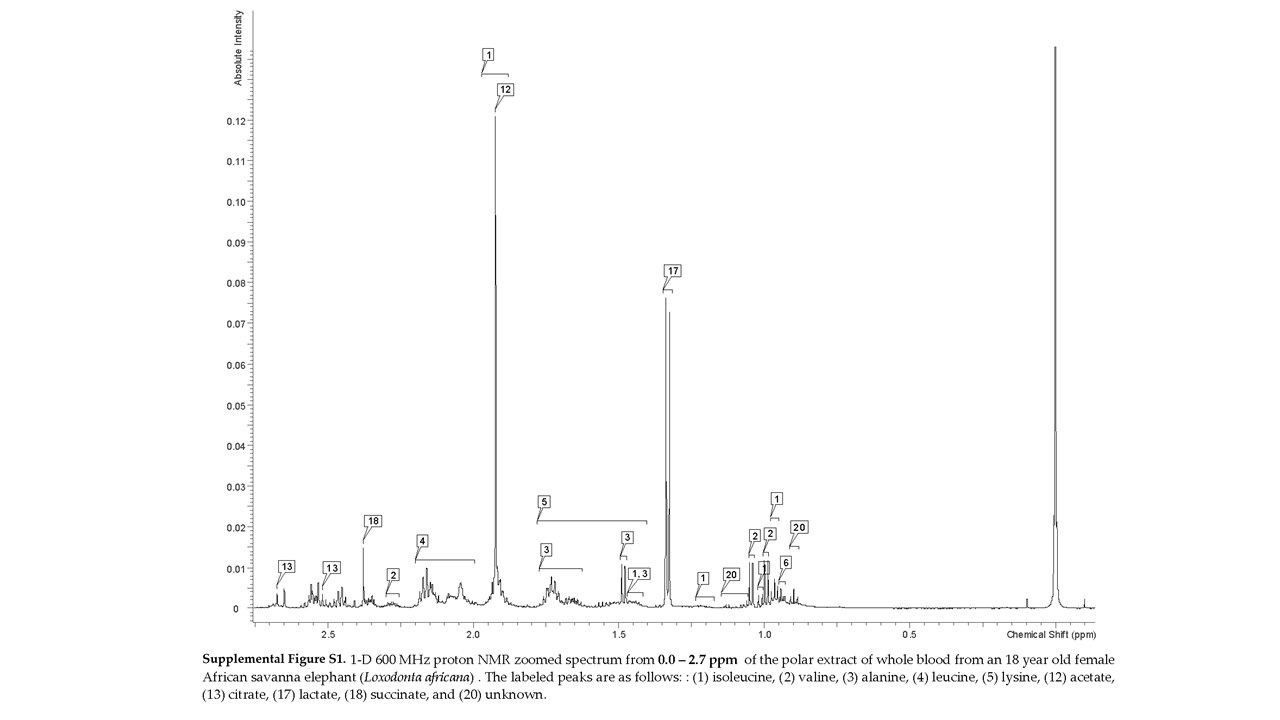

Supplement: Supplementary file 1 [file metabolites-12-00400-s001.zip › Supplemental Figure S1.tif]

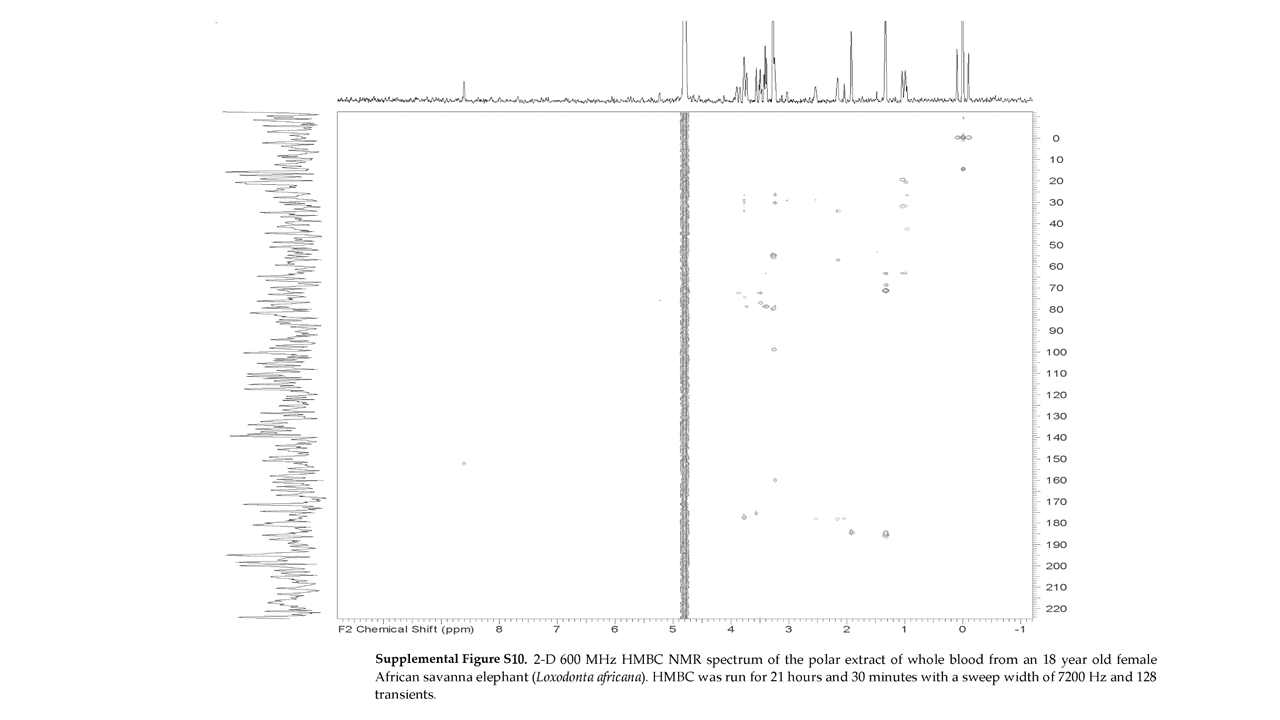

Supplement: Supplementary file 1 [file metabolites-12-00400-s001.zip › Supplemental Figure S10.tif]

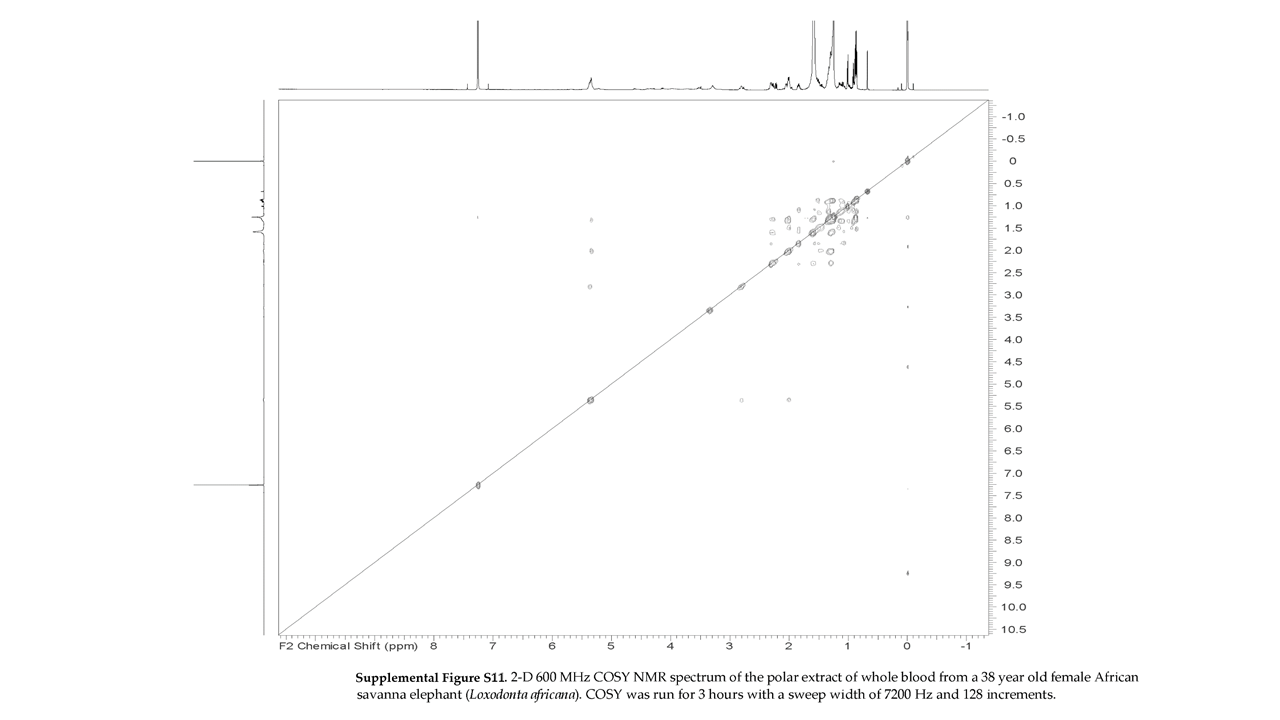

Supplement: Supplementary file 1 [file metabolites-12-00400-s001.zip › Supplemental Figure S11.tif]

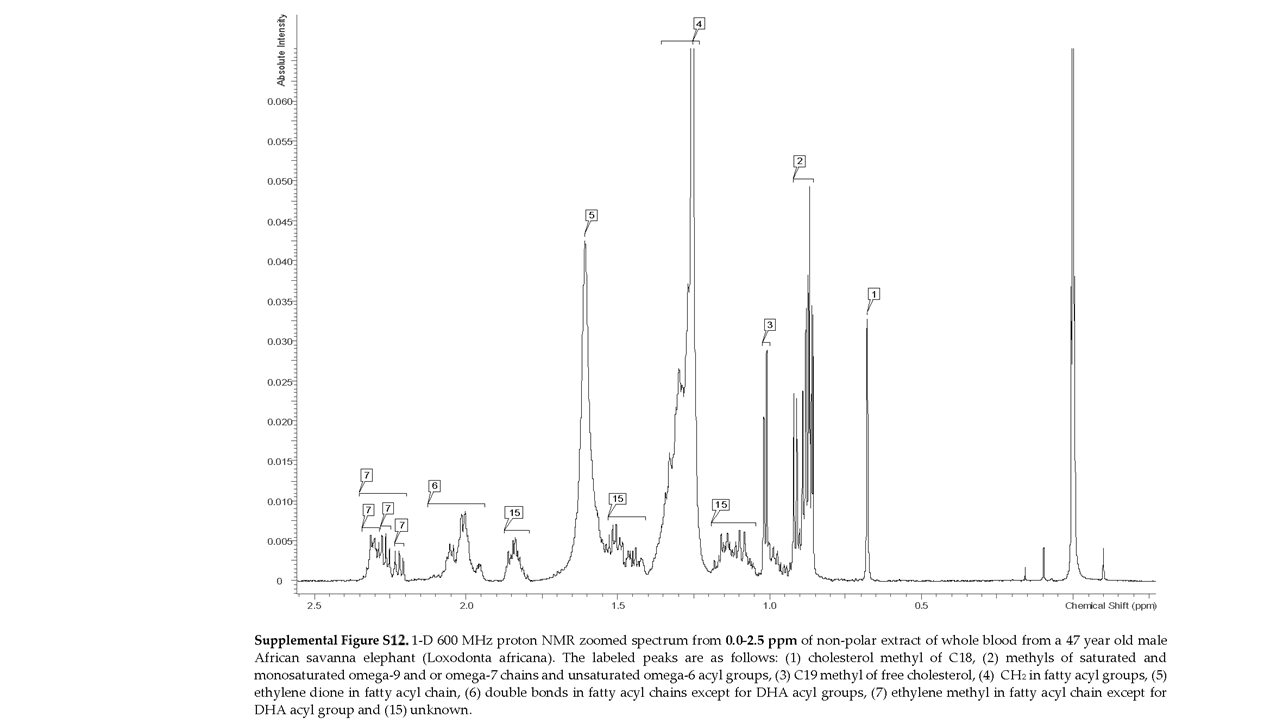

Supplement: Supplementary file 1 [file metabolites-12-00400-s001.zip › Supplemental Figure S12.tif]

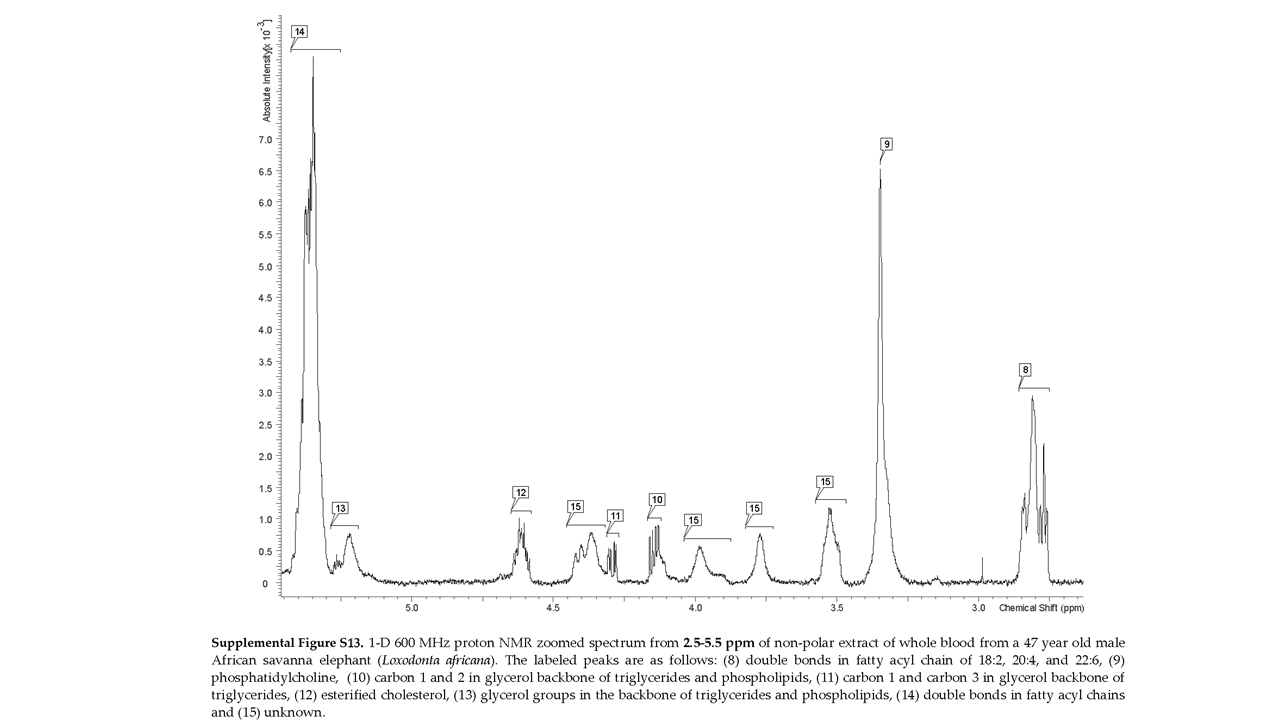

Supplement: Supplementary file 1 [file metabolites-12-00400-s001.zip › Supplemental Figure S13.tif]

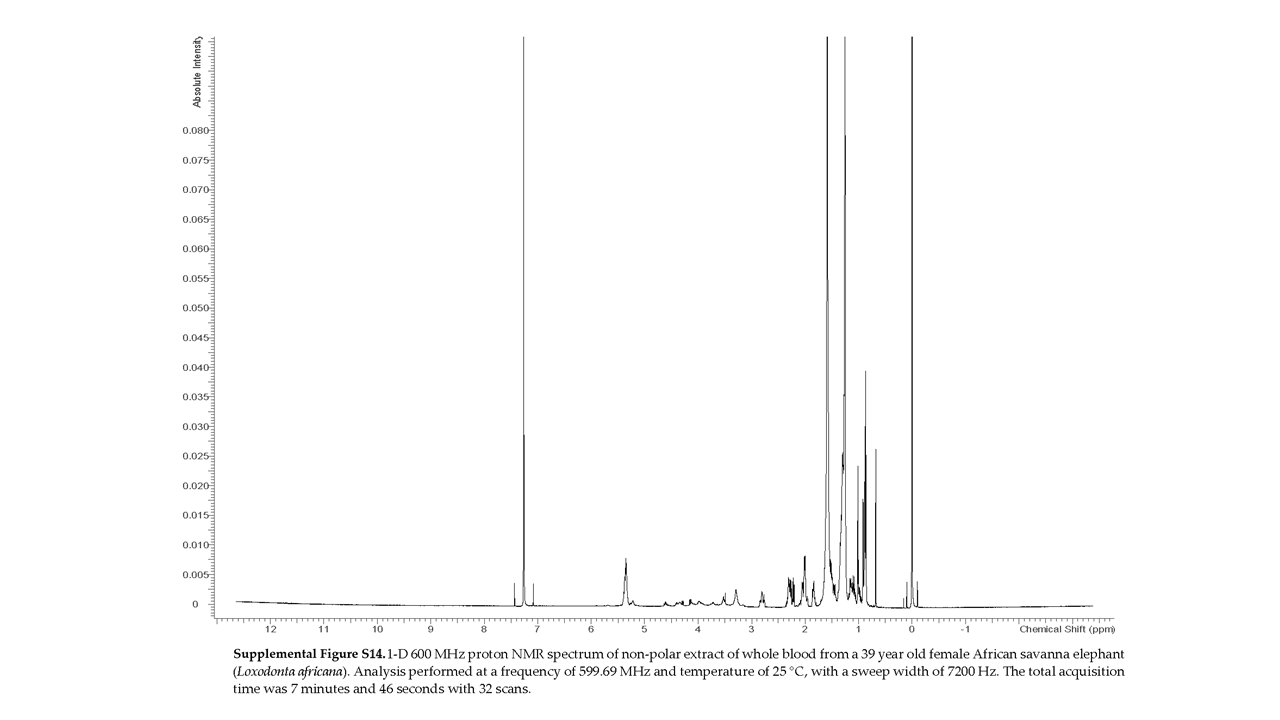

Supplement: Supplementary file 1 [file metabolites-12-00400-s001.zip › Supplemental Figure S14.tif]

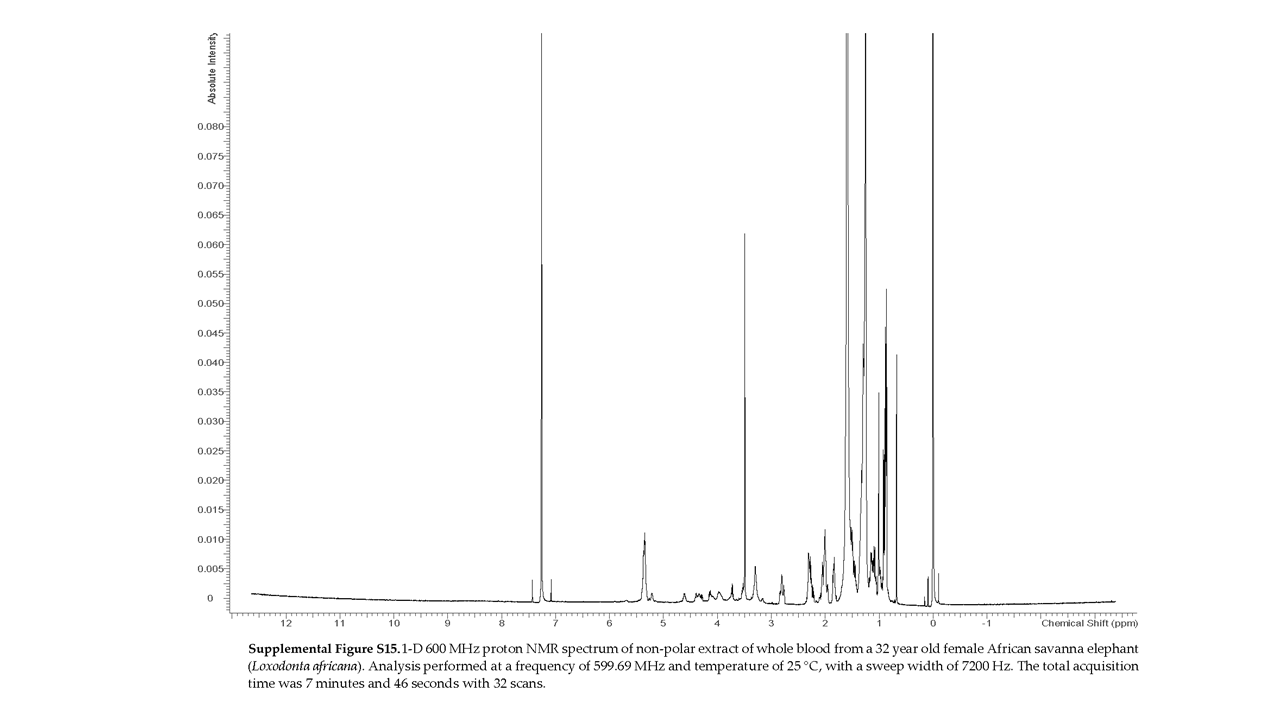

Supplement: Supplementary file 1 [file metabolites-12-00400-s001.zip › Supplemental Figure S15.tif]

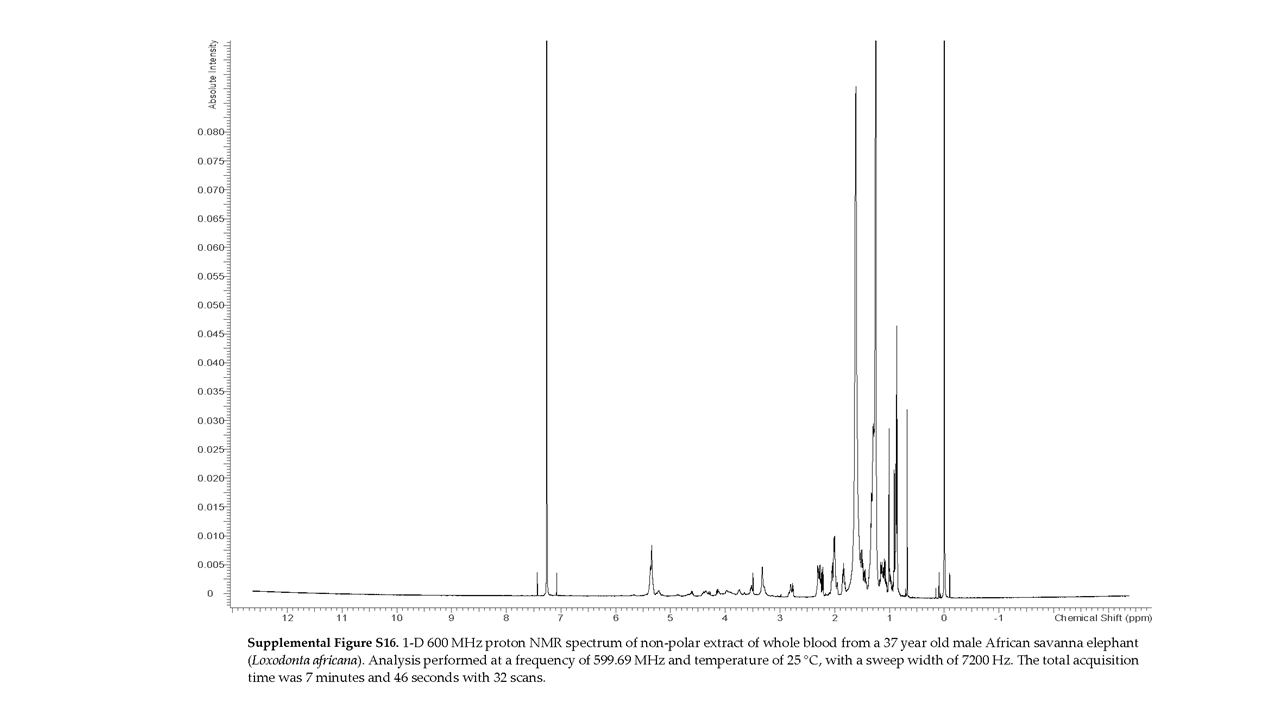

Supplement: Supplementary file 1 [file metabolites-12-00400-s001.zip › Supplemental Figure S16.tif]

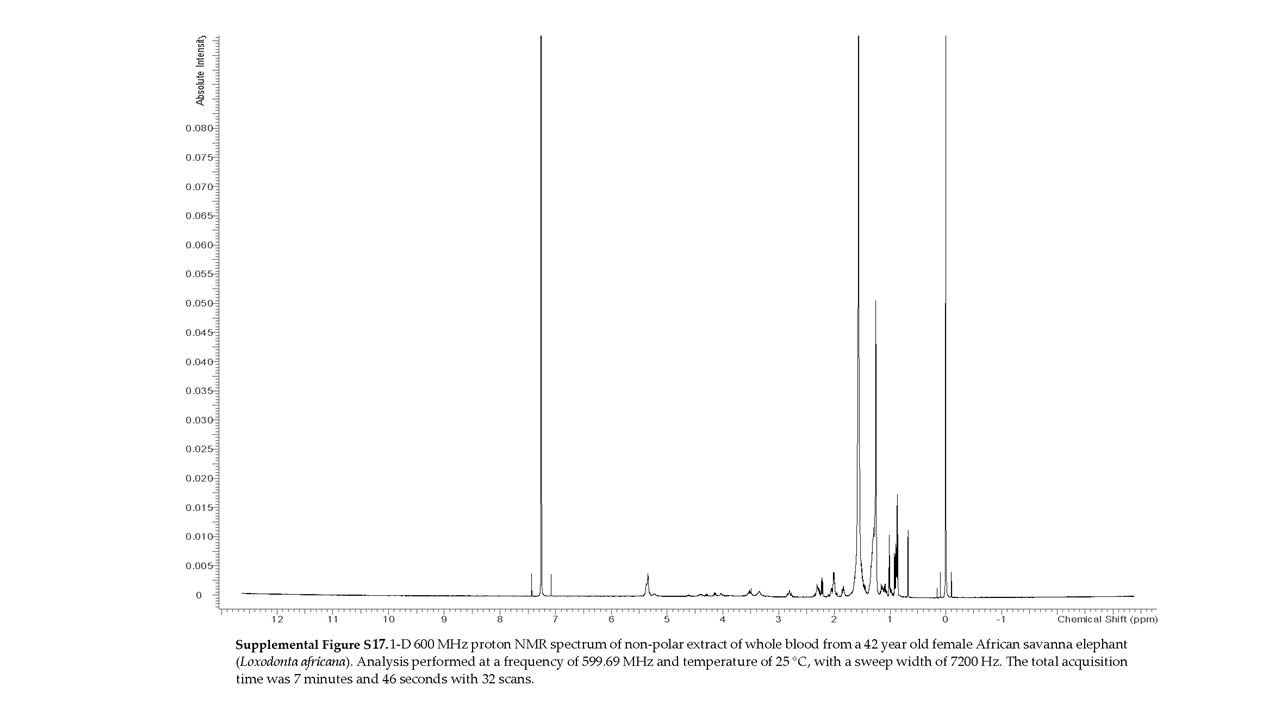

Supplement: Supplementary file 1 [file metabolites-12-00400-s001.zip › Supplemental Figure S17.tif]

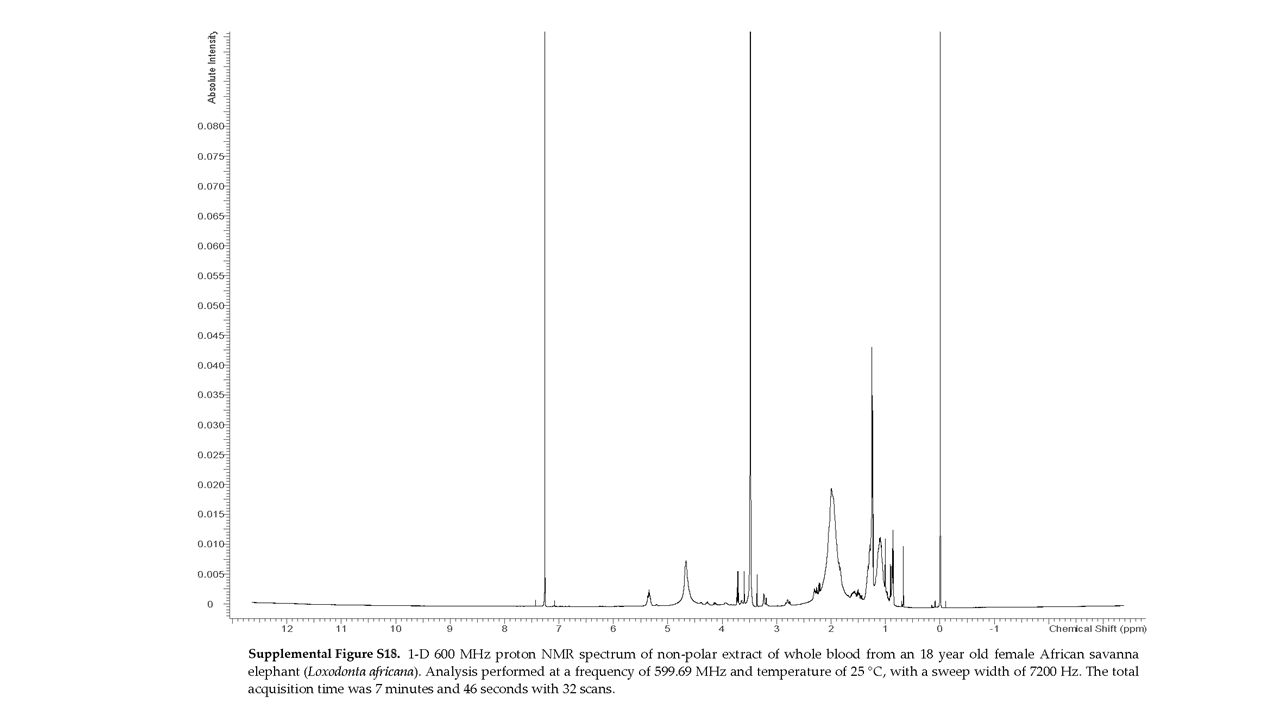

Supplement: Supplementary file 1 [file metabolites-12-00400-s001.zip › Supplemental Figure S18.tif]

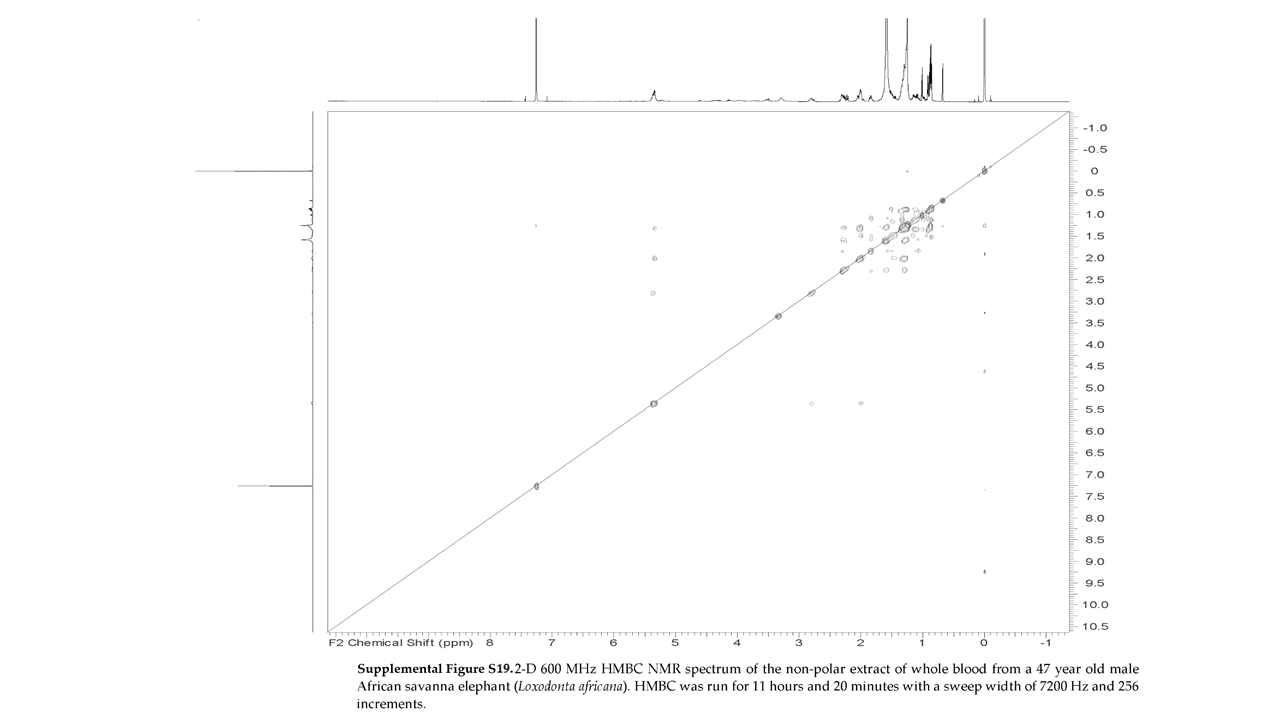

Supplement: Supplementary file 1 [file metabolites-12-00400-s001.zip › Supplemental Figure S19.tif]

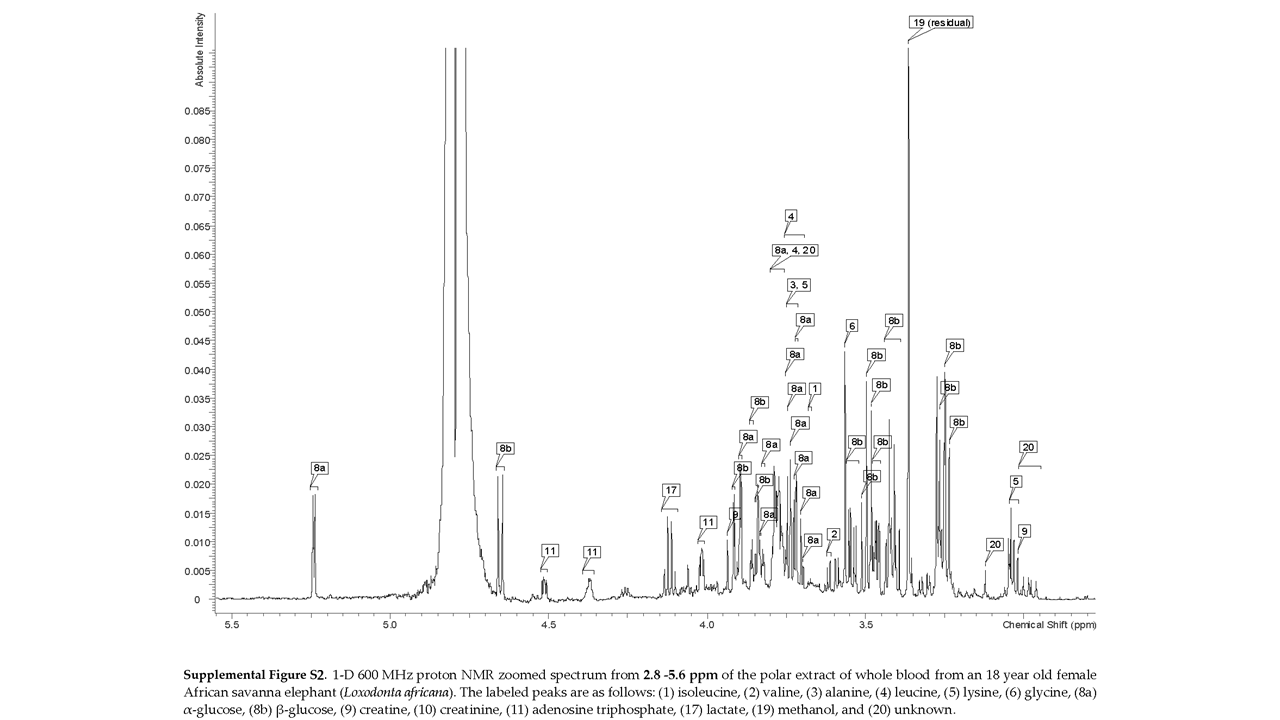

Supplement: Supplementary file 1 [file metabolites-12-00400-s001.zip › Supplemental Figure S2.tif]

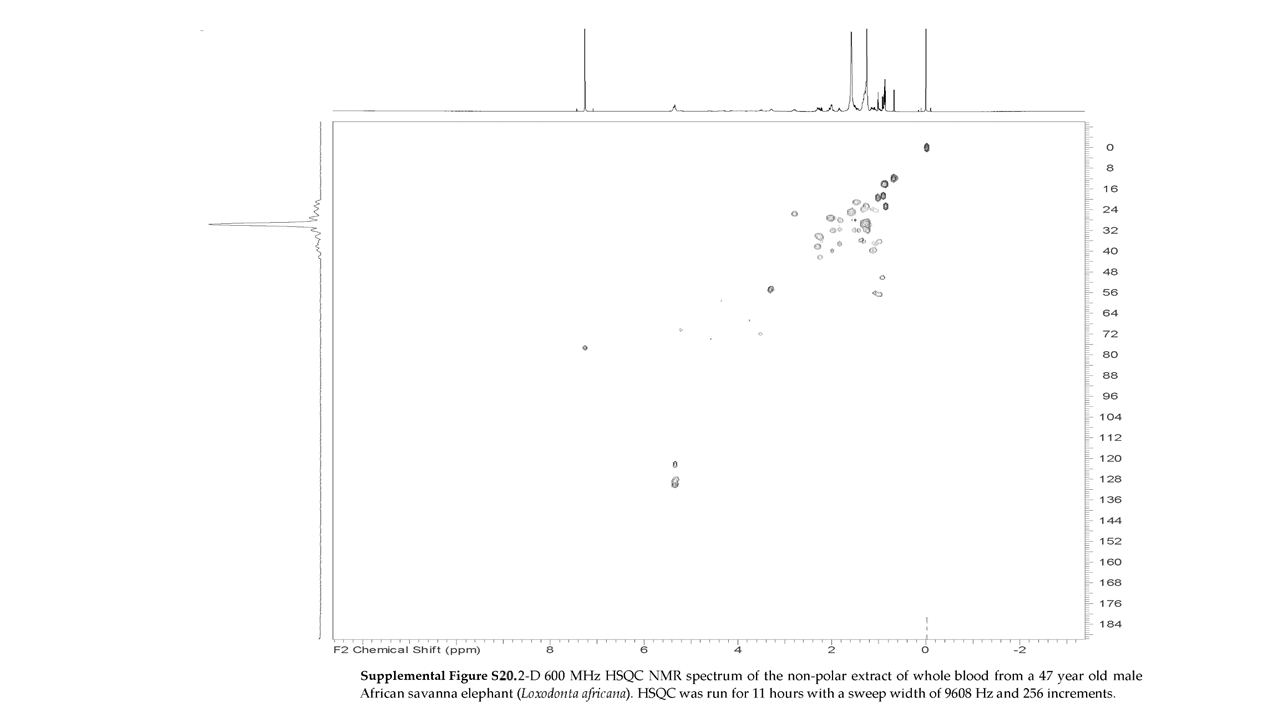

Supplement: Supplementary file 1 [file metabolites-12-00400-s001.zip › Supplemental Figure S20.tif]

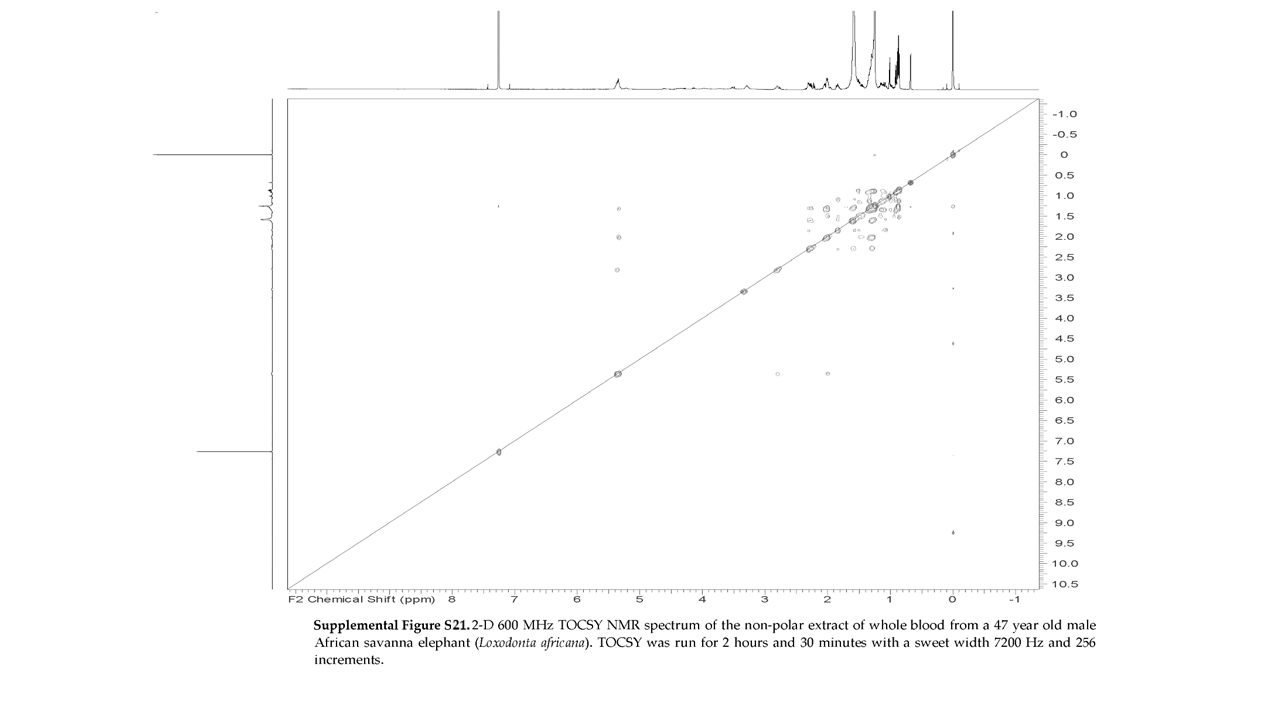

Supplement: Supplementary file 1 [file metabolites-12-00400-s001.zip › Supplemental Figure S21.tif]

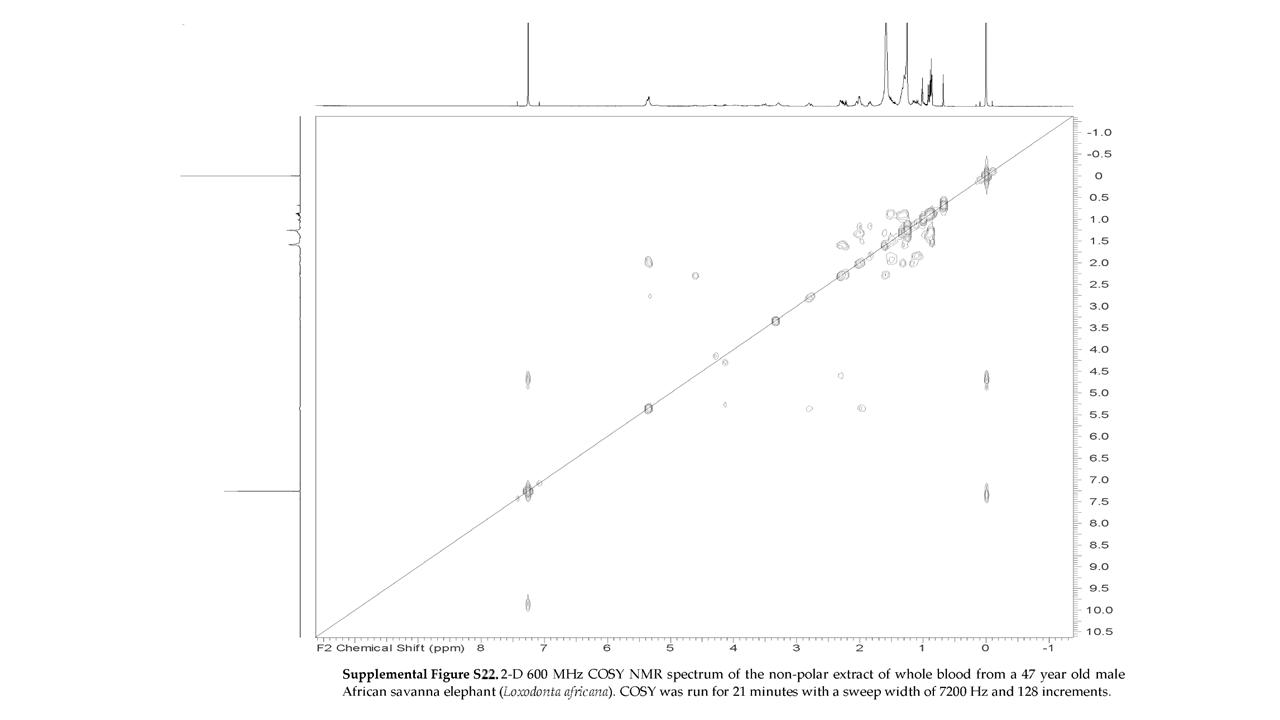

Supplement: Supplementary file 1 [file metabolites-12-00400-s001.zip › Supplemental Figure S22.tif]

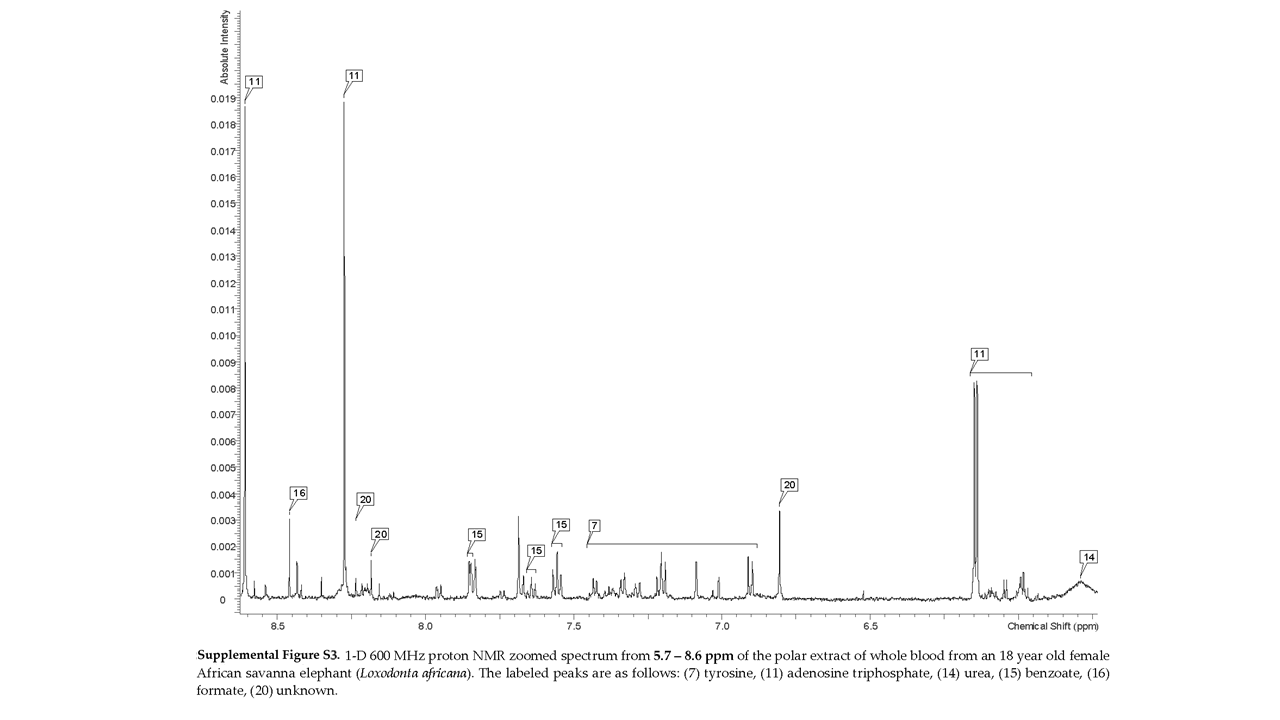

Supplement: Supplementary file 1 [file metabolites-12-00400-s001.zip › Supplemental Figure S3.tif]

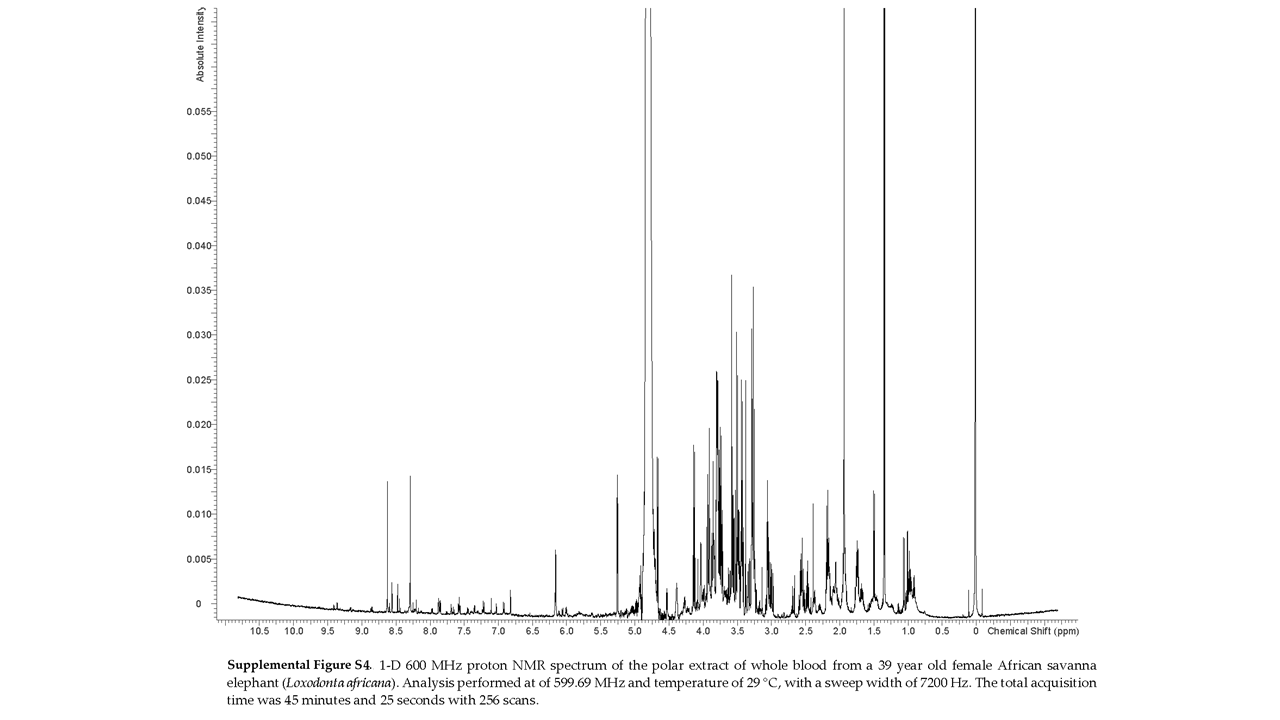

Supplement: Supplementary file 1 [file metabolites-12-00400-s001.zip › Supplemental Figure S4.tif]

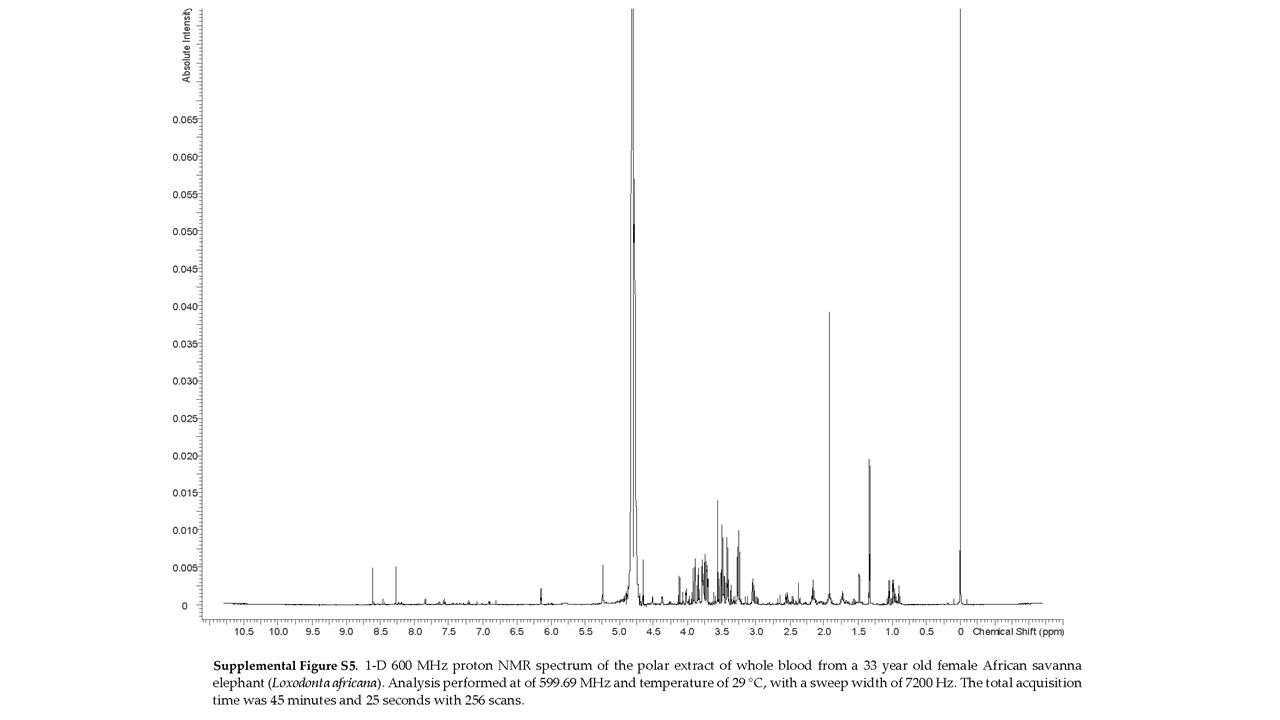

Supplement: Supplementary file 1 [file metabolites-12-00400-s001.zip › Supplemental Figure S5.tif]

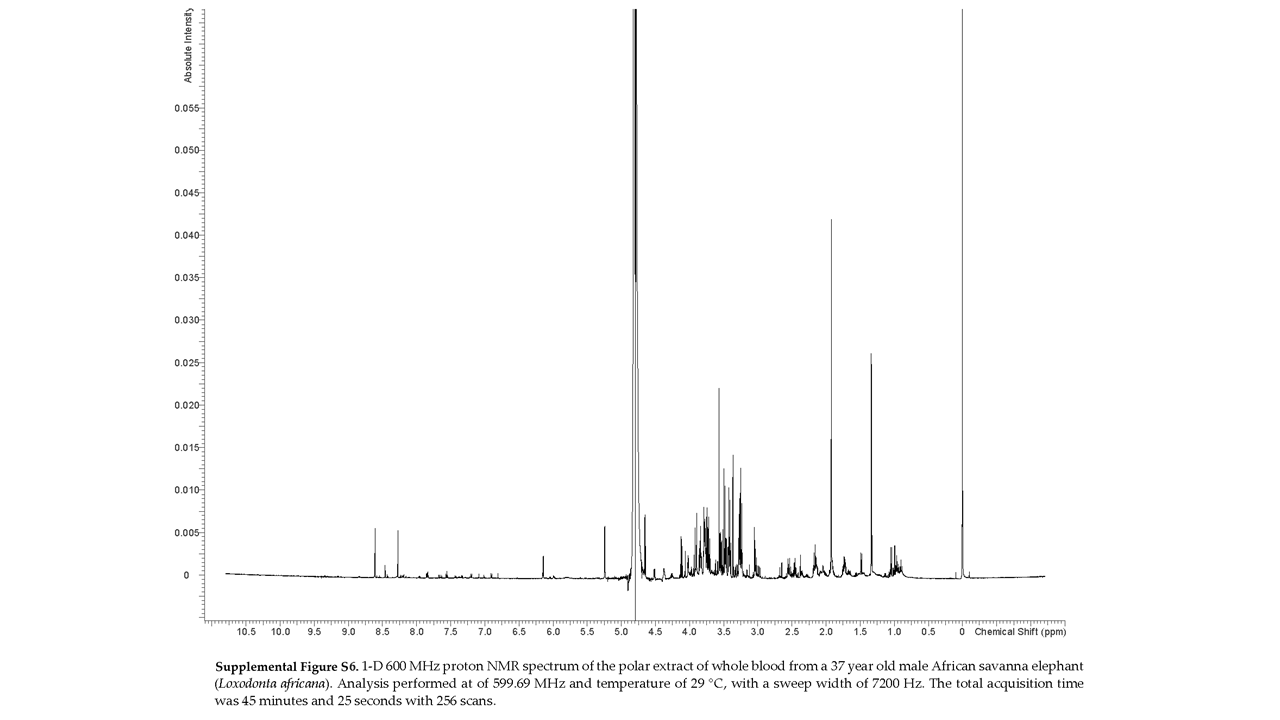

Supplement: Supplementary file 1 [file metabolites-12-00400-s001.zip › Supplemental Figure S6.tif]

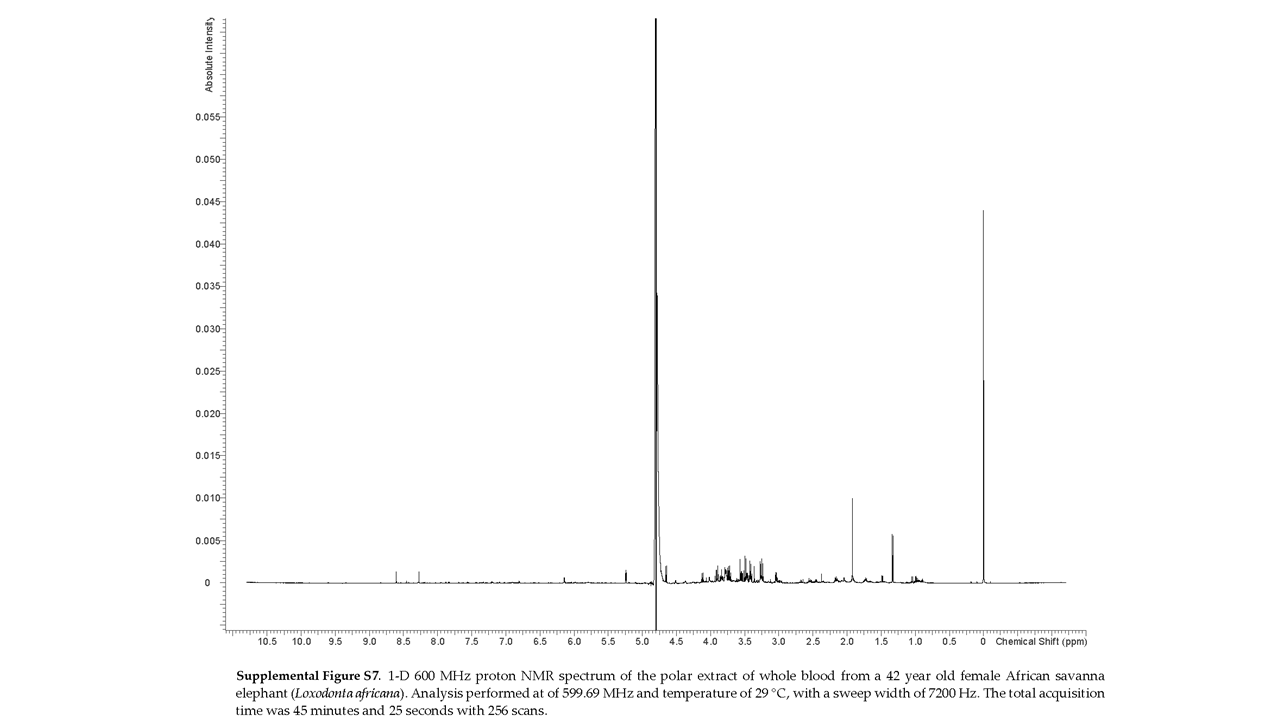

Supplement: Supplementary file 1 [file metabolites-12-00400-s001.zip › Supplemental Figure S7.tif]

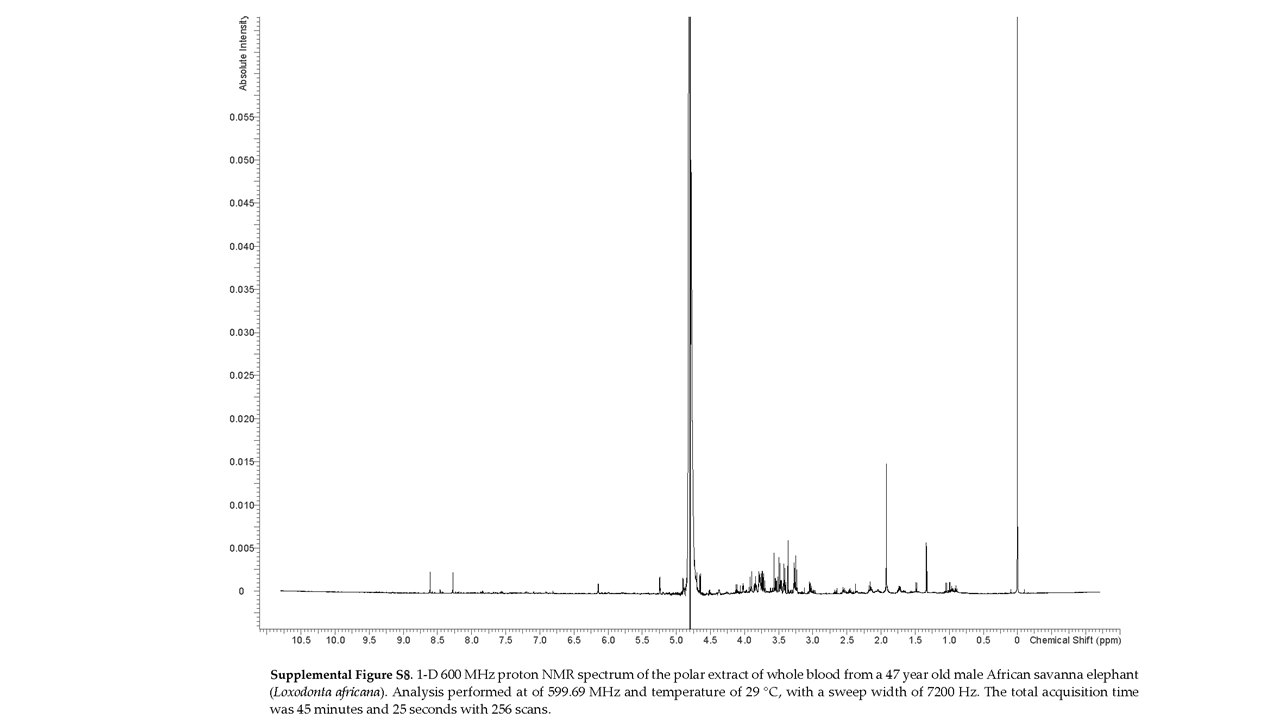

Supplement: Supplementary file 1 [file metabolites-12-00400-s001.zip › Supplemental Figure S8.tif]

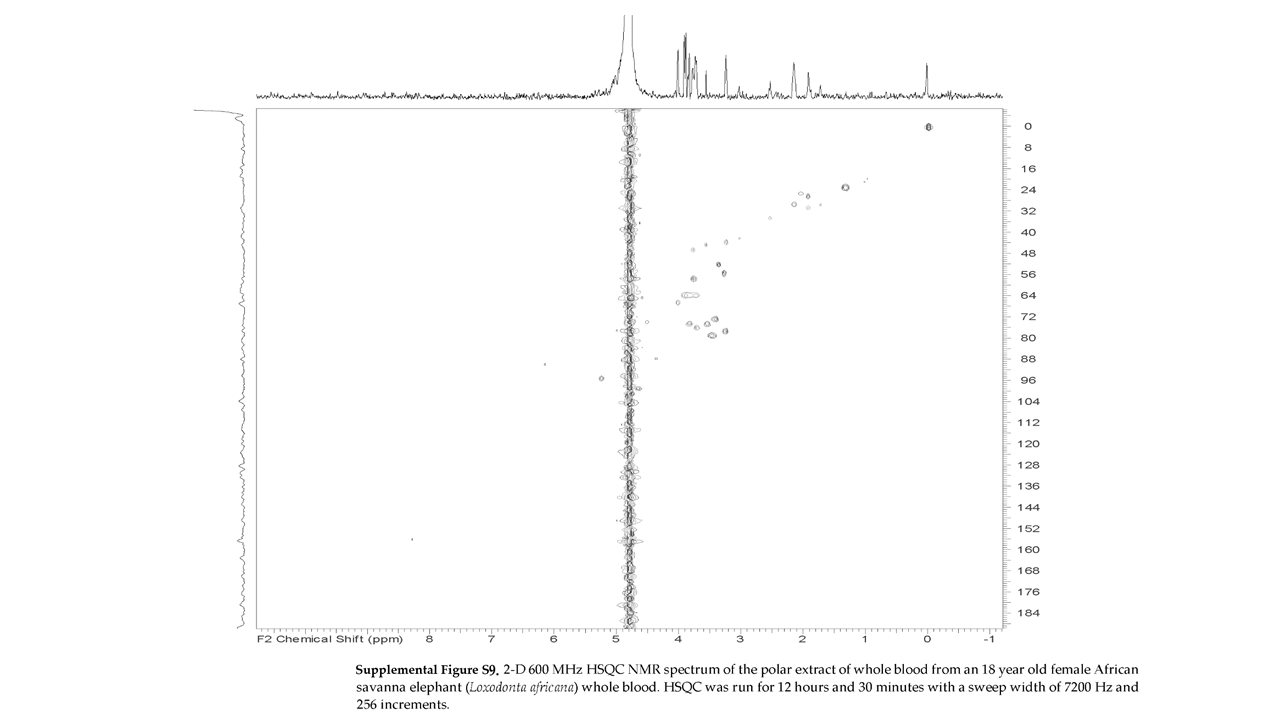

Supplement: Supplementary file 1 [file metabolites-12-00400-s001.zip › Supplemental Figure S9.tif]

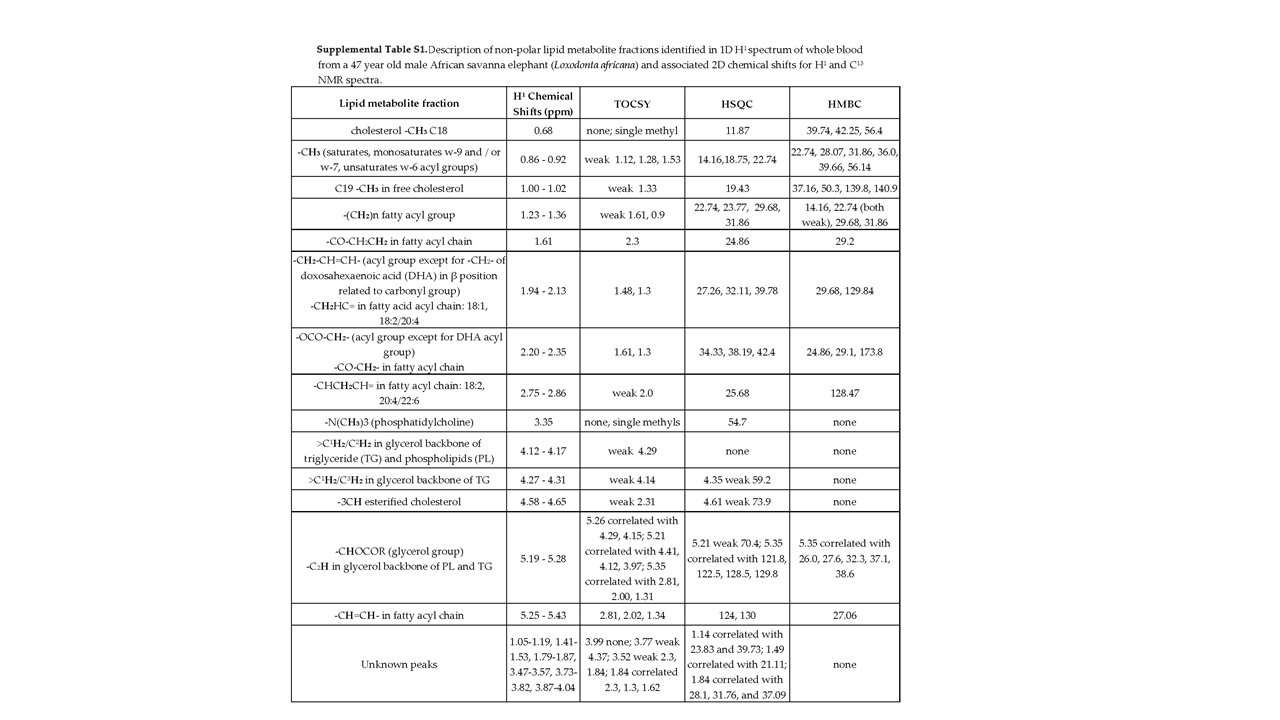

Supplement: Supplementary file 1 [file metabolites-12-00400-s001.zip › Supplemental Table S1.tif]
